# Critical factors associated with postpartum maternal death in Ethiopia

**DOI:** 10.1371/journal.pone.0270495

**Published:** 2022-06-24

**Authors:** Neamin Tesfay, Rozina Tariku, Alemu Zenebe, Fitsum Woldeyohannes

**Affiliations:** 1 Centre of Public Health Emergency Management, Ethiopian Public Health Institutes, Addis Ababa, Ethiopia; 2 Health Financing Program, Clinton Health Access Initiative, Addis Ababa, Ethiopia; Jawaharlal Institute of Postgraduate Medical Education and Research, INDIA

## Abstract

**Background:**

Globally most maternal deaths occur during the postpartum period; however, the burden is disproportionately higher in some Sub-Saharan African countries including Ethiopia. According to Ethiopian Ministry of Health’s annual report, in 2019 alone, nearly 70% of maternal deaths happen during the postpartum period. Although several studies have been conducted on postpartum maternal deaths in Ethiopia, most of the studies were focused either on individual-level or district-level determinants with limited emphasis on the timing of death and in relatively small and localized areas. Therefore, this study aimed at identifying the determinants of postpartum death both at an individual and districts level, which could shed light on designing pragmatic policies to reduce postpartum maternal death.

**Methods:**

The study utilized secondary data obtained from the Ethiopian maternal death surveillance system. A total of 4316 reviewed maternal death from 645 districts of Ethiopia were included in the analysis. A multilevel multinomial logistic regression model was applied to examine factors significantly associated with postpartum maternal death in Ethiopia.

**Result:**

The findings revealed that 65.1% of maternal deaths occurred during the postpartum period. The factors associated with postpartum death included previous medical history (history of ANC follow up and party), medical causes (obstetrics haemorrhage, hypertensive disorder of pregnancy, pregnancy-related infection, and non-obstetrics complication), personal factors (poor knowledge of obstetrics complication), and facility-level barriers (shortage of life-saving maternal commodities and delay in receiving treatment).

**Conclusion:**

Almost seven in ten maternal deaths happen during the postpartum period. The rate was even higher for some women based on their previous medical history, level of awareness about obstetrics complication, medical conditions, as well as the readiness of the health facility at which the women was served. Since the postpartum period is identified as a critical time for reducing maternal death, policies and actions must be directed towards improving health education, ANC service utilization, and facility-level readiness.

## Introduction

Maternal death is the death of a woman while pregnant or within 42 days of termination of pregnancy [1]. Globally, between 2000 to 2017, the maternal mortality rate (MMR) dropped by 38% from 451,000 to 295,000. Despite this great reduction in MMR, it remains significantly high in low- and middle-income countries. Sub-Saharan African countries account for approximately 60% of the estimated global maternal deaths in 2017 [2]. To address this and other global development gaps, a new global strategy has been set in 2015 called the Sustainable Development Goal (SDG) which was aimed to replace the Millennium Development Goal (MDGs) that culminated in 2015. The SDG considers maternal mortality as one of the global health indicators and sets a target to reduce global maternal mortality to fewer than 70 deaths per 100,000 live births by 2030 [3].

In Ethiopia, the absolute number of maternal deaths has decreased by 55% from 31,000 in 1990 to 14,000 in 2017 [2, 4]. The country has made tangible strides in reducing maternal mortality in the last two decades. The MMR has declined from around 871 deaths in 2000 to 401 deaths in 2017; despite this achievement, there is noticeable variation among regional states [5–7]. Due to these regional variations the country has failed to achieve one of the MDGs goals, which had a target of dropping MMR to 267 in 2015 [4].

Intending to provide real-time data on the patterns and trends of preventable maternal death, Ethiopia established maternal death surveillance and response system (MDSR) in 2013 [8]. MDSR is a continuous process of identifying maternal deaths, gathering information on their cause and determinants, and analyzing the data to avert similar deaths in the future [9]. Moreover, using the evidence generated, different measures are being taken in different settings. At a health facility level, the actions are aimed at addressing provider-related barriers (skills, medical supplies, and referral mechanisms) [10], while at a higher level the focus is on interpreting patterns and trends in maternal deaths. It also facilitates decisions on targeting resources to reduce inequities and improve public awareness and health-seeking behaviour [11]. In line with this, maternal death has become one of the compulsory notifiable events under the Ethiopian public health surveillance system [12].

Along with the establishment of MDSR, Ethiopia has launched various initiatives to reduce preventable maternal death, which include the construction of maternity waiting rooms (MWRs) around health facilities [13, 14], utilization of non-pneumonic anti-shock garments (NASG) during referral [15, 16], and availability of free transport and maternity services [17]. However, despite all these efforts and interventions, MMR remains to be unacceptably high in Ethiopia [18].

Understating the timing of maternal death has paramount importance in planning health programs and setting priorities [19]. The timing of maternal death has three-time windows classified based on labour and delivery. These are; 1) death that happened before the onset of labour (antepartum death), 2) death that happened during the onset of labour (intrapartum death), and 3) death occurring after delivery (postpartum death) [20].

Globally, 57% of maternal deaths occur after the delivery, i.e during the postpartum period, however, there is a notable variation across countries [20, 21]. Among the postpartum deaths, the majority of the maternal deaths occur within 24 hours of childbirth, followed by death within seven days of delivery and from two to six weeks after childbirth, each accounting for 50%, 20%, and 5% of maternal mortality, respectively [22]. Similarly, in Ethiopia, most maternal deaths occurred during the postpartum period with an estimated range from 51% to 75% of the total reported deaths [23–25].

Research findings have demonstrated that most maternal deaths occur during the postpartum period [20, 21, 25]. Postpartum maternal death has a connection with maternal service provision during the antepartum and intrapartum periods [26]. The maternal services include ANC visits, which play an integral role in screening, diagnosing, and managing potential maternal and child health threats such as anemia, malaria, worm infestation, HIV, and pre-eclampsia [27]. Besides, it has a pivotal contribution to safe delivery [28]. Similarly, intrapartum care also has a crucial role in maternal health, and it has a direct relation to facility-level readiness such as diagnosis of labour, ensure of clean delivery techniques, early detection, and referral of maternal complications [29]. The utilization and provision of both services have a cumulative impact on postpartum maternal death, and it is related to the quality of maternity service provision [30]. The quality of maternal service is largely dependent on the availability of trained personnel, essential maternal health commodities, and physical infrastructure [31]. The combination of factors such as service provision during the antepartum and intrapartum period in line with the quality of care makes the postpartum period the critical time for maternal survival and wellbeing [32].

Overall, the timing of maternal death is influenced by medical causes and contributing factors [33–35]. Haemorrhage, hypertensive disorder of pregnancy (HDP), severe anaemia unrelated to haemorrhage, and obstetric infection are the commonest cause of maternal death during the postpartum period [36–38]. However, medical factors are not the only factors that determine postpartum maternal death, it could as well be affected by facility-level readiness [39, 40], referral-related barriers [41, 42], and birth preparedness and complication plan of the women [43].

Several studies were conducted on maternal death in Ethiopia; however, none of the studies used both individual factors (medical factors and level of birth preparedness) [44–47], and facility-level factors (on barriers related to referral and services provision) [48, 49] simultaneously. In addition, most of the studies were conducted in a small geographical area with limited emphasis on postpartum maternal death. Nevertheless, the timing of maternal death is influenced by both individual and community-level factors. Thus, this study aims to identify the determinants of postpartum maternal death both at an individual and facility-level among reviewed maternal deaths across Ethiopia.

## Methods

### Study setting

This study is conducted in Ethiopia, which is in the horn of Africa. The country’s population is generally categorized as city-based population, pastoralists, and agrarian. Ethiopia is one of the most populous countries in the continent, with a total population of more than 114 million, of which 34% (above 39 million) are women of the reproductive age group [50]. Most of the population, around 83%, lives in rural areas, while the remaining 17% resides in urban areas. The average household size in Ethiopia is 4.7 persons, although with notable variations among regions [51]. The country has a high neonatal, infant, and child mortality rate, averaging 30, 43, and 55 deaths per 1000 livebirth, respectively [52].

### Data source and study participant

The study used data from Ethiopian Public Health Institutes (EPHI). It utilized an updated programmatical and epidemiological review of maternal death data obtained from all MDSR implementing regions for seven consecutive years (2013–2020).

The source population for this study is all mothers who were deceased due to pregnancy and related complications and reviewed by the MDSR committee during the study period. Accordingly, a total of 4316 reviewed maternal death were included in the study. The death review was conducted by the established maternal death review committee at each health facility within the 645 districts of Ethiopia.

### Death identification and reporting

#### Population under surveillance

All women of the reproductive age group in Ethiopia are eligible for the surveillance system.

Case definition

The surveillance data is reported to the next level using pre-defined case definitions implemented in facility and community settings [53].

- **Community case definition** (probable maternal deaths): Death of a woman of reproductive age group (between 15–49 years of age)
- **Suspected maternal deaths:** Community case definition plus at least one of the following screening questions
  ✓ Died while pregnant
  ✓ Died within 42 days of termination of pregnancy or
  ✓ Missed her menses before she died
- **Standard case definition**: Death of a woman while pregnant or within 42 days of the end of pregnancy (Irrespective of duration and site of pregnancy), from any cause related to or aggravated by the pregnancy or its management but not from accidental or incidental causes [53, 54].

#### Investigation and verification of death

After the death is distinguished using the above case definitions, it proceeds to the next step, which is the investigation and verification of death by the health extension workers at the community level; while, at a health facility level, public health emergency management (PHEM) focal person is responsible. Following that, a verbal autopsy is utilized to investigate and verify the community death. The investigation only proceeds after taking verbal consent from the family of the deceased woman. However, the facility-based abstraction format (FBAF) is utilized for facility deaths [53].

#### Review of death

Each completed verbal autopsy and facility-based maternal death abstraction must go through the review process by an established MDSR committee at the health facility. After the review, the death is reported to the next level using a case-based reporting format [53].

### Study variables

#### Outcome variables

The dependent variable is the time of death relating to delivery. The multinomial dependent variable was classified as “Antepartum” (decreased before delivery), “Intrapartum” (decreased during delivery), and “Postpartum” (decreased after delivery).

#### Exposure variables

The selection of potential predictors of the time of death was based on previous literature and the availability of variables in reviewed case-based reports. An attempt was made to test all potentially relevant variables that existed on the nationally aggregated maternal death data before including/excluding them in the final model. Accordingly, all variables which showed a statistical significance or some sort of trend during bivariate analysis were included in the analyses. Potential predictors were then categorized into two groups: individual-level predictors and district-level predictor variables.

Individual factors such as age at death, education level, religion, history of antenatal follow-up, parity, place of death, the medical cause of death (MCOD), and the non-medical cause of death (only delay one) were included in the study. The MCOD was assigned using the standard world health organization (WHO) tool of the International statistical classification of diseases and related health problems, the tenth revision (ICD-10), which was adopted for deaths during pregnancy, childbirth, and the puerperium [55]. The assigned cause of death for each reviewed maternal death was grouped based on underlying causes of death during pregnancy, childbirth, and the puerperium which were mutually exclusive from one another. Moreover, delay one, which is associated with delay in seeking care, was measured by 5 item questioners that include: 1) visited a traditional healer or traditional birth attendant first 2) the family had insufficient money 3) lack of awareness of obstetric complications 4) nearest healthcare facility was more than 1 km away 5) lack of decision to health facility due to perceived poor quality of care at health service. Responses to delay one questions were binary recoded “1” for Yes and “0” for No.

District-level factors include residence (urban and rural), contextual regions, delay two, and delay three. The 11 regions of Ethiopia are delineated for administrative purposes, and in this study, they were re-categorized into three contextual regions: pastoralist, agrarian, and city (which were defined based on the cultural and socio-economic backgrounds of their population) [56]. Delay two, which is indicative of delay in reaching a healthcare facility, was assessed using 5 item questions that include: 1) poor road condition or terrain 2) long travel time from home to a healthcare facility (more than an hour) 3) cost of transportation 4) lack of transportation and 5) no healthcare facility in the area (takes more than one hour to reach the healthcare facility). Furthermore, delay three, which is suggestive of delay in receiving care at the healthcare facility, was assessed using 4 item questions: 1) long travel time from health facility to health facility (which is usually related to inadequate referral system described through unavailability of ambulances, lack of fuel, technical failure of the ambulance while in service, and the use of public transport) 2) shortage of equipment and supplies 3) delay in management (service provision) after admission (more than 30 min from the time of arrival to the time of being assessed or receiving treatment) and 4) wrong assessment of risk, wrong diagnosis, and treatment.

### Data analysis

#### Descriptive analysis

Descriptive statistics were conducted for the individual and district-level variables and are reported as frequency and percentage. Furthermore, to examine the crude association between the individual and community-level factors separately with a distinct time of death, p-values were calculated using Pearson’s chi-squared test. A p-value of less than 0.05 was set for the statistical significance of an association.

#### Multivariate multilevel analysis

Two-level mixed-effects logistic regression analyses were employed using STATA version 17. The surveillance data was hierarchical, i.e., deceased women were nested in reporting facilities, and similarly reporting facilities were nested in districts. As a result of the nature of the data, mothers within the same district may be more similar to each other than mothers in the rest of the country. By considering the clustering effect, initially, a bivariate two-level mixed-effects logistic regression analysis was done to assess the association between the independent variables and the dependent variable of the study. The overall categorical variables with a p-value of <0.25 at the bivariate two-level mixed-effect logistic regression analysis were included in the final model of the multivariate two-level mixed-effects multinomial logistic regression model, in which the relative risk ratio(RRR) with 95% confidence intervals were estimated to identify independent variables of time of death of the deceased women [57]. P-values less than 0.05 were employed to declare statistical significance. Fixed effect and random effect were calculated to assess the individual and district variations, respectively. For the multilevel multinomial logistic regression analysis, the Stata ‘gsem’ syntax was employed [58]. Thus, four models were utilized in this analysis, the empty model (model containing no factors), Model I (containing only individual factors), Model II (containing only districts factors), and Model III (both individual and district-level factors). The fitted model was:

$$\log\left[y_{ij}-s/p(y_{ij}-postpartum)\right]-X\beta^{\left(s\right)}+u_{oj}^{\left(s\right)},$$

where,

yij is the time of death for the deceased woman I who resided in district J.

S is the outcomes (antepartum, intrapartum, and postpartum).

X is the matrix of independent variables at both individual and district levels.

β(s) is the effect size of each independent variable on the probability of women dying during antepartum and intrapartum

μoj(s)is a constant term and cross-level interaction term.

The intra-class correlation (ICC) was calculated as the proportion of the between cluster variation in the total variation:

ICC=Var(Uoj)Var(Uoj)+π23

Where Var (Uoj) is community-level variance and π23 is individual-level variance (VI) equal to 3.29. The ICC takes a value between 0 and 1 and a high ICC value indicates that neighbourhoods are important in understanding individual differences in postpartum [59]. The MOR is defined as the median value of the odds ratio between the area at highest risk and the area at the lowest risk when randomly picking out two areas and it depends directly on the area-level variance [60]. It can be calculated using the following formula:

$$MOR=(exp\sqrt{2\;X\;Var(Uoj)X\;0.6745)}\approx exp\;(0.95\sqrt{Var(Uoj)}$$

Where Var (Uoj) is the district-level variance, and 0.6745 is the 75th centile of the cumulative distribution function of the normal distribution with mean 0 and variance 1. In this study, MOR shows the extent to which the individual probability of experiencing death during postpartum is determined by the residential area [59]. The PCV is used to measure the total variation attributed to individual-level factors and area-level factors in the multilevel model [59, 61]. It was calculated using the following mathematical equation:

$$PCV=\frac{Ve-Vmi}{Ve}$$

Where Ve is the variance in antepartum and intrapartum in the empty model and Vmi is the neighbourhood variance in the subsequent model.

*Model fit statistics*. Akaike’s information criterion (AIC) and Schwarz’s Bayesian information criteria (BIC) were used to assess the goodness of fit and inform the selection of nested models (individual- and community-level models). The AIC and BIC values were compared in successive models and the model with the lowest value was selected as a best-fit model [62].

#### Ethical issues

Ethical approval was obtained from the Ethical Review Committee and Public Health Emergency.

Management unit of Ethiopian Public Health Institute (EPHI) with Ref. No. EPHI 4_1/37. To keep confidentiality, personal identifiers were not used in the study. Since the study used secondary data sources, consent and other ethical measures were not applicable.

## Result

### Selected background characteristics of maternal death reviewed facilities

The proportion of death during the postpartum period was estimated at 65.1%, during the antepartum period was 19.8% and during the intrapartum period was 15.0%. Among reported facilities, more than half (58.3%) of the deaths were reported from primary-level health care facilities, and 98.8% from public facilities. More than half (57.9%) of the reviewed maternal deaths were reported from Amhara and Oromia regions. Moreover, nearly half (49.3%) of the deaths were reviewed in 2016 and 2017, (Table 1).

**Table 1:** Selected background characteristics of reporting facilities by the time of death in Ethiopia, 2020.

Variable/category	Time of death			Total	P-value
	Antepartum n (%)	Intrapartum n (%)	Postpartum n (%)		
**Type of health facility**					
Primary level health care	489(19.4)	415(16.5)	1614(64.1)	2518	0.001
Secondary level health care	235(18.6)	170(13.4)	861(68.0)	1266	
Tertiary level heath care	132(19.8)	64(15.0)	336(65.2)	532	
**Ownership of facility**					
Private	2(16.7)	1(8.3)	9(75.0)	12	0.875
NGO	8(21.1)	4(10.5)	26(68.4)	38	
Government	846(19.8)	644(15.1)	2776(65.1)	4266	
**Reporting region**					
Tigray	97(17.2)	76(13.4)	393(69.4)	566	0.0001
Afar	28(45.2)	7(11.3)	27(43.5)	62	
Amhara	249(20.6)	179(14.8)	779(64.6)	1207	
Oromia	205(15.8)	235(18.1)	855(66.1)	1295	
Somali	3(12.5)	4(16.7)	17(70.8)	24	
Ben-Gum	24(30.8)	8(10.2)	46(59.0)	78	
SNNPR	139(25.0)	81(14.6)	335(60.4)	555	
Gambella	5(16.1)	5(16.1)	21(67.8)	31	
Harari	29(33.7)	7(8.1)	50(58.2)	86	
Addis Ababa	31(8.2)	42(33.7)	176(58.1)	249	
Dire Dawa	35(21.5)	16(9.8)	112(68.7)	163	
**Year of reporting**					
2013	2(15.4)	2(15.4)	9(69.2)	13	0.002
2014	38(12.8)	72(24.4)	186(62.8)	296	
2015	91(18.5)	93(19.0)	307(62.5)	491	
2016	156(18.8)	143(17.3)	529(63.9)	828	
2017	249(19.1)	209(16.1)	843(64.8)	1301	
2018	133(19.5)	93(13.6)	457(66.9)	683	
2018	133(19.5)	93(13.6)	457(66.9)	683	
2020	48(21.1)	15(6.6)	164(72.2)	227	

### Sociodemographic characteristics of the deceased women

The proportion of women who died during the postpartum period was higher among women aged between 40–49 years (71.6%) compared to women who were aged between 10–19 years (60.2%). Women who resided in a rural area had a higher proportion of death during intrapartum (15.6%) compared to those who resided in an urban area (11.6%). The proportion of women who died during the antepartum period was higher among women with parity between 0 and 1 (22.2%) compared to those who had a parity above five (16.3%), (Table 2).

**Table 2:** Distribution of personal characteristics by time of death among reviewed maternal death in Ethiopia, 2020.

Variable/category	Time of death			Number	significant
	Antepartum(n) (%)	Intrapartum(n) (%)	Postpartum(n) (%)		
**Age group**					
10_19	60(24.4)	38(15.4)	148(60.2)	246	0.029
20_29	425(20.2)	294(14)	1382(65.8)	2101	
30_39	330(19.1)	289(16.7)	1107(64.1)	1726	
40_49	41(16.9)	28(11.5)	174(71.6)	243	
**Residence area**					
Urban	132(20.5)	75(11.6)	438(67.9)	645	0.032
Rural	724(19.7)	574(15.6)	2373(64.6)	3671	
**Place of death**					
On transit	116(19.7)	114(19.4)	358(60.9)	588	0.001
Home	117(15.6)	85(11.3)	550(73.1)	752	
Health facility	623(20.9)	450(15.2)	1903(63.9)	2976	
**Marital status**					
Unmarried	65(22.8)	39(13.6)	182(63.6)	286	0.41
Married	791(19.6)	610(15.2)	2629(65.2)	4030	
**Religion**					
Traditional	5(14.7)	8(23.5)	21(61.8)	34	0.303
Muslim	336(20.9)	247(15.4)	1021(63.7)	1604	
Christian	515(19.2)	394(14.7)	1769(66.1)	2678	
**Educational status**					
Secondary and above	60(17.0)	58(16.4)	235(66.6)	353	0.18
Primary	78(17.0)	78(17.0)	303(66.0)	459	
Illiterate	718(20.5)	513(14.6)	2273(64.9)	3504	
**Parity**					
0–1	335(22.2)	228(15.1)	945(62.7)	1508	0.001
2_4	342(20.0)	269(15.7)	1100(64.3)	1711	
5	179(16.3)	152(13.9)	766(69.8)	1097	
**History of ANC follow up**					
Yes	254(18.4)	168(12.1)	962(69.5)	1384	0.001
No	602(20.5)	481(16.4)	1849(63.1)	2932	

### The proportion of assigned cause of death for reviewed maternal death

The proportion of women who died during the postpartum period was higher among women deceased due to pregnancy-related infection (81.5%) as compared to women who died due to other causes of death. Women who died due to unanticipated complications of management had a higher proportion of death during the intrapartum period (25.0%) compared to women who died due to other causes. The proportion of women who died due to abortive outcomes of pregnancy was higher among women who died during the antepartum period (77.1%) as compared to the remaining cause of death, (Table 3).

**Table 3:** Distribution of cause of death by time of death among reviewed maternal death in Ethiopia, 2020.

Variable/category	Time of death			Number	significant
Cause of death	Antepartum n (%)	Intrapartum n (%)	Postpartum n (%)		
Coincidental cause					
Yes	2(66.7)	0(0.0)	1(33.3)	3	0.12
No	854(19.8)	649(15.0)	2810(65.2)	4313	
Unanticipated complication of management					
Yes	15(25.0)	15(25.0)	30(50.0)	60	0.031
No	841(19.8)	634(14.9)	2781(65.3)	4256	
Other obstetrics complication					
Yes	24(18.9)	29(22.8)	74(58.3)	127	0.043
No	856(19.9)	649(14.8)	2811(65.3)	4316	
Abortive pregnancy outcome					
Yes	74(77.1)	2(2.1)	20(20.8)	96	0.001
No	782(18.5)	647(15.3)	2791(66.1)	4220	
Unknown /undetermined					
Yes	51(22.6)	47(20.8)	128(56.6)	226	0.12
No	805(19.7)	602(14.7)	2683(65.6)	4090	
Pregnancy-related infection					
Yes	45(12.8)	20(5.7)	286(81.5)	351	0.001
No	811(20.5)	629(15.9)	2525(63.7)	3965	
Non-obstetrics complication					
Yes	163(35.2)	46(10.0)	253(54.8)	462	0.001
No	693(18.0)	603(15.6)	2558(66.4)	3854	
Hypertensive disorder of pregnancy					
Yes	160(26.4)	85(14.0)	361(59.6)	606	0.01
No	696(18.8)	564(15.2)	2450(66.0)	3710	
Obstetric haemorrhage					
Yes	322(13.5)	405(17.0)	1658(69.5)	2385	0.001
No	534(27.7)	244(12.6)	1153(59.7)	1931	

### The proportion of contributing causes of death

The proportion of women who died during the postpartum period was much higher among women who were wrongly diagnosed and treated (72.9%) compared to other contributing factors. Women who died due to lack of transportation had a higher proportion of death during intrapartum (21.6%) compared to other contributing factors. The proportion of women who died due to lack of money for transportation was higher among women who were deceased during the antepartum period (25.6%) as compared to delay factors, (Table 4).

**Table 4:** Distribution of contributing causes of death by time of death among reviewed maternal death in Ethiopia, 2020.

Contributing factor	Time of death			Number	P-value
	Antepartum n (%)	Intrapartum n (%)	Postpartum n (%)		
Delay -1(decision to seek care)					
Traditional practices					
Yes	133(18.6)	97(13.5)	486(67.9)	716	0.229
No	723(20.1)	552(15.3)	2325(64.6)	3600	
Family poverty (Low status)					
Yes	65(21.1)	47(15.3)	196(63.6)	308	0.821
No	791(19.7)	602(15.1)	2615(65.2)	4008	
Lack of awareness of obstetric complications					
Yes	270(23.3)	138(11.9)	749(64.8)	1157	0.001
No	586(18.6)	511(16.1)	2062(65.3)	3159	
Failed to decide to go to a health facility					
Yes	296(19.5)	215(14.1)	1010(66.4)	1521	0.364
No	560(20.1)	434(15.5)	1801(64.4)	2795	
Long-distance to a healthcare facility					
Yes	256(20.5)	203(16.2)	792(63.3)	1251	0.234
No	600(19.6)	446(14.6)	2019(65.8)	3065	
Delay 2(reaching care)					
Poor road condition or terrain					
Yes	62(20.0)	57(14.8)	218(65.2)	337	0.536
No	794(20.0)	592(14.8)	2593(65.2)	3979	
Long travel time from home to a healthcare facility					
Yes	206(20.0)	151(14.6)	674(65.4)	1031	0.921
No	650(19.7)	498(15.2)	2137(65.1)	3285	
Lack of money for transport					
Yes	33(25.6)	20(15.5)	76(58.9)	129	0.221
No	823(19.7)	629(15.0)	2735(65.3)	4187	
Lack of transportation					
Yes	95(16.6)	124(21.6)	355(61.8)	574	0.001
No	761(20.3)	525(14.1)	2456(65.6)	3742	
No healthcare facility in the area					
Yes	32(16.0)	30(15.0)	138(69.0)	200	0.361
No	824(20.1)	619(15.0)	2673(64.9)	4116	
Delay 3(receiving care)					
Long travel time from HF to HF፟					
Yes	208(21.8)	166(17.4)	578(60.8)	952	0.004
No	648(19.2)	483(14.4)	2233(66.4)	3364	
Shortage of equipment and supplies					
Yes	88(19.6)	48(10.7)	313(69.7)	449	0.019
No	768(19.9)	601(15.5)	2498(64.6)	3867	
Long waiting time before treatment was received					
Yes	81(15.9)	69(13.6)	359(70.5)	509	0.019
No	775(20.4)	580(15.2)	2452(64.4)	3807	
Wrong assessment of risk, wrong diagnosis, wrong treatment					
Yes	40(15.3)	31(11.8)	191(72.9)	262	0.024
No	816(20.2)	618(15.2)	2620(64.6)	4054	

### Factors associated with an antepartum time of death in Ethiopia among reviewed deaths

Women who died in transit were more likely to die during the antepartum period compared to those who died at home [RRR = 1.94,95% CI:(1.38–2.71)]. Women who had a parity of more than five were less likely to die during the antepartum period compared to those who were nulliparous [RRR = 0.74; 95% CI:(0.58–0.94)]. Women who died as a result of pregnancy-related infection were less likely to die during the antepartum period compared to those who did not die due to pregnancy-related infection [RRR = 0.18, 95% CI:(0.110.31)]. A similar finding was observed in women who were deceased due to abortive outcome of pregnancy, obstetrics haemorrhage, HDP, and non-obstetrics complications. Women who failed to recognize the danger signs of pregnancy and those who were managed lately after admission were less likely to die during the antepartum period compared to those who recognized the danger sign or were treated immediately after admission, respectively [RRR = 0.78, 95% CI:(0.62,0.97)] for danger sing and [RRR = 0.33, 95% CI: (0.170.61)] for a time of treatment). Women who were not treated well due to a shortage of equipment and supplies were less likely to die during the antepartum period as compared to women who were treated well with the necessary supplies and equipment [RRR = 0.63, 95% CI: (0.45–0.88)]. The risk of death during the antepartum period was significantly higher among women who travelled long distances to reach a health facility or does not get transportation to a health facility compared to those who did not travel a long distance to reach a health facility or those who have access to transportation, respectively [RRR = 1.59, 95% CI:(1.23–2.04)] for long-distance and [RRR = 1.30–95% CI:(1.05–1.61)] for transportation), (Table 5).

**Table 5:** Multilevel multinomial analysis of individual and community factors associated with the time of death among reviewed maternal death in Ethiopia, 2020.

Variables/Characteristics	Empty model	Model 2^b^		Model 3^c^		Model 4^d^	
		Individual characteristics		Community characteristics		Individual and Community characteristics	
	Post-partum^(a)^	Antepartum	Intrapartum	Antepartum	Intrapartum	Antepartum	Intrapartum
		RRR (95%CI)	RRR (95%CI)	RRR (95%CI)	RRR (95%CI)	RRR (95%CI)	RRR (95%CI)
**Death of place**							
Home®		1	1			1	1
On transit		2.17(1.56,3.01) ***	1.73(1.26,2.38) **			1.94(1.38,2.71) ***	1.64(1.19,2.27) **
Health Facility		1.69(1.28,2.22) ***	1.50(1.17,1.93) **			1.76(1.33,2.34) ***	1.47(1.14,1.90) **
**Parity**							
0–1®		1	1			1	1
5 and above		0.78(0.62,1.00) *	0.71(0.57,0.89) *			0.74(0.58,0.94) *	0.71(0.56,0.89) **
2–4		1.01(0.82,1.25)	0.90(0.74,1.09)			0.98(0.80,1.21)	0.88(0.73,1.08)
**History of ANC follow up**							
No®		1	1			1	1
Yes		0.66(0.53,0.82) ***	0.77(0.64,0.94) **			0.68(0.55,0.84) ***	0.77(0.64,0.94) ***
**Abortive outcome of pregnancy**							
No®		1	1			1	1
Yes		0.29(0.07,1.30)	11.00(6.13,19.75) ***			0.29(0.07,1.29)	10.79(6.01,19.37) **
**HDP**							
No®		1	1			1	1
Yes		0.60(0.42,0.85) ***	1.05(0.76,1.46)			0.58(0.41,0.83) **	1.02(0.74,1.42)
**Obstetrics hemorrhage**							
No®		1	1			1	1
Yes		0.60(0.45,0.79) ***	0.48(0.36,0.64) ***			0.57(0.42,0.75) ***	0.47(0.35,0.62) ***
**Pregnancy-related infection**							
No®		1	1			1	1
Yes		0.18(0.11,0.31) ***	0.39(0.26,0.59) ***			0.18(0.11,0.31) ***	0.37(0.25,0.57) ***
**None- obstetrics complication**							
No®		1	1			1	1
Yes		0.49(0.32,0.73) **	1.66(1.19,2.31) **			0.48(0.32,0.73) **	1.61(1.15,2.24) **
**Failure to recognize the complication**							
No®		1	1			1	1
Yes		0.78(0.63,0.97) *	1.24(1.03,1.50) *			0.78(0.62,0.97) *	1.23(1.02,1.49) *
**Regional_Residence**							
City administration				1	1	1	1
Pastoralist				1.34(0.70,2.54)	1.80(1.06,3.06) *	1.50(0.78,2.88)	1.92(1.10,3.34) *
Agrarian				1.51(1.00,2.28) *	0.97(0.67,1.4)	1.47(0.96,2.25)	1.1(0.75,1.61)
**Lack of transportation**							
No®				1	1	1	1
Yes				1.55(1.22,1.97) ***	0.82(0.64,1.06)	1.59(1.23,2.04) ***	0.94(0.72,1.23)
**Long travel time from home to a healthcare facility**							
No®				1	1	1	1
Yes				1.41(1.14,1.74) **	1.30(1.07,1.57) **	1.30(1.05,1.61) **	1.32(1.08,1.62) **
**Shortage of equipment and supplies**							
No®				1	1	1	1
Yes				0.64(0.46,0.89) **	0.88(0.68,1.15)	0.63(0.45,0.88) *	0.84(0.63,1.11)
**Delayed management after admission**							
No®				1	1	1	1
Yes				0.93(0.70,1.23)	0.75(0.57,0.97) *	0.87(0.65,1.70)	0.71(0.54,0.95) *

*P < 0.05

**P < 0.001

***P < 0.0001

^(a)^ Reference for the dependent variable

® Reference for the category of an independent variable

### Factors associated with an intrapartum time of death in Ethiopia among reviewed deaths

Women who died on transit [RRR = 1.64, 95%CI:(1.19–2.27)] or in health facility [RRR = 1.47,95% CI:(1.14–1.19)] were more likely to die during intrapartum period compared to women deceased at home. Similarly, women deceased due to abortive outcome of pregnancy [RRR = 10.79, 95% CI:(6.01–19.37)] and non-obstetrics complication [RRR = 1.61,95% CI: (1.15,2.24)]; who failed to recognize the danger signs [RRR = 1.23; 95% CI: (1.02–1.49)] and who resided in pastoralist region [RRR = 1.92, 95% CI: (1.10–3.34)] were more likely to die during intrapartum. The odds of dying during the intrapartum period were much lower among grand para women [RRR = 0.71, 95% CI: (0.56–0.89)], women with history of ANC follow-up [RRR = 0.77, 95% CI: (0.64–0.94)] and women treated lately after admission [RRR = 0.71, 95% CI:(0.54–0.95)]. Moreover, women deceased due to obstetrics hemorrhage [RRR = 0.47, 95% CI: (0.35–0.62)] and pregnancy related infection [RRR = 0.37; 95% CI: (0.25,0.57)] were less likely to die during the intrapartum period, (Table 5).

#### Multilevel analysis (random-effects analysis)

Table 6 presents quantities based on random effects. The status of time of death varied across districts (τ^2^ = 0.47, p = <0.001). The empty model revealed that 12.3% of the total variance in time of death was accounted for by between-cluster variation of characteristics (ICC = 0.123). The district variability declined over successive models, from 12.3% in the empty model to 10.6% in the individual-level only model, 9.9% in the districts-level only model, and 9.6% in the final (combined) model. The proportional change in variance indicated that the addition of predictors to the empty model explained an increased proportion of variation in the time of death. Similar to ICC values, the combined model showed a higher PCV, i.e., 46% of the variance in the status of time of death could be explained by the combined factors at the individual and community levels. Furthermore, the MOR confirmed that the time of death was attributed to district-level factors. The MOR for a time of death was 1.90 in the empty model, which indicated the presence of variation between districts for a time of death since MOR was nearly two times higher than the reference (MOR = 1). The unexplained districts’ variation for time of death decreased to a MOR of 1.75 when all factors were added to the empty model. This indicates that 0.25 (25%) of the heterogeneity was explained by both individual and community level factors, but still, there is a residual effect that is not explained by individual and district-level variables at the final full model (MOR = 1.75). This implies that even though individual- and district-level factors were considered, the effect of clustering is still statistically significant in the full model.

**Table 6:** Results from the random intercept model (a measure of variation) for the timing of death at the district level using multilevel logistic regression analysis.

Random effect	Model_1^a^	Model_2^b^	Model_3^c^	Model_4^d^
District level variance (SE)	0.47(0.11)	0.39(0.08)	0.36(0.08)	0.35(0.07)
P_values	<0.001	<0.001	<0.001	<0.001
ICC (%)	12.3%	10.6%	9.9%	9.6%
Explained variance (PVC) (%)	Reference	15.2%	21.1%	46.0%
MOR (95%CI)	1.90(1.68,2.27)	1.81(1.63,2.07)	1.76(1.58,2.01)	1.75(1.60,2.04)
Model fit statics				
AIC	7500	7153	7512	7111
BIC	7591	7216	7551	7206

SE = Standard Error; DIC = Deviance Information Criterion; ICC = Intra-Class Correlation; PCV = Percentage Change in Variance; MOR = Median Odds Ratio; CI = Confidence Interval; AIC = Akaike’s Information Criterion; BIC = Schwarz’s Bayesian Information Criteria.

Model_1^a^ is the empty model, a baseline model without any determinant variable

Model_2^b^ is adjusted for individual-level factors

Model_3^c^ is adjusted for community-level factors

Model_4^d^ is the final model adjusted for the individual- and community-level factors

#### Model fit statistics

As shown in Table 6 (model fit statistics), the values of AIC and BIC showed subsequent reduction which indicates each model represents a significant improvement over the previous model and it points to the goodness of fit of the final model built in the analysis.

## Discussion

The study observed the hierarchal effect of various factors that determine the timing of death among reviewed maternal deaths in Ethiopia. Our study has demonstrated that the timing of maternal death is determined by both individual factors (previous medical history, medical cause, and knowledge of obstetrics complications) and districts level factors (lack of transportation, shortage of life-saving maternal commodities in a health facility, and delay in receiving treatment).

Women’s parity was positively associated with postpartum maternal death. This finding was comparable with studies done in Tanzania [63], Nigeria [64], Saudi Arabia [65], and India [66]. The possible justification might be related to pre-existing health conditions of the women, as well as access and utilization of maternity services [67, 68] and the combination of these factors may affect the outcome of the mother after delivery. In agreement with this, women of advanced maternal age, who are believed to have a relatively high parity are at an increased risk of maternal death. Per the recent Ethiopian demographic and health survey, only 18%,36%, and 40% of women of advanced maternal age used contraceptives; attended more than 4 ANC visits; and delivered at a health facility, respectively [52]. These findings imply that more improvement in the access and utilization of maternal service is particularly needed for multipara women. This should be coupled with adequate service provision to reduce maternal death during the postpartum period.

History of ANC visit was also one of the individual-level factors significantly associated with postpartum maternal death. Women who had at least one ANC visit were less likely to die during the antepartum and intrapartum periods. In line with this, the study also revealed that nearly 70% of the women were deceased at health facilities after delivery. This partly explains that attending ANC service encourages institutional delivery; however, it is not a guarantee for obtaining quality maternity service [69]. The uptake of the service may be compromised by various factors including the educational status of the women, the residence of the women, distance from the facility, and previous history of successful home delivery [70–73]. This suggests that the integration of ANC services with other maternal services and programs should be a priority to meet the ultimate objective of the ANC service. Meanwhile, improvement in the managing capacity of obstetrics emergencies should be the other priority to reduce postpartum maternal death significantly.

Among individual factors, the medical cause of death—both direct (HDP, obstetrics haemorrhage, and pregnancy-related infection) and indirect (non-obstetrics complication)–were positively associated with maternal death after delivery. The finding was concurrent, in the case of obstetrics haemorrhage, with studies conducted in Ethiopia (Debere-Tabore, Yirgalem, and Harir) [74–76], Nigeria [77], South Africa [78], and Mozambique [79]. The possible reason might be related to the management of the third phase of labour, which is provided by a trained health professional at a health facility. Ethiopia has put in place various measures to tackle the burden of obstetrics haemorrhage. Some of the measures that were put in place include the utilization of NASG during referral [80], provision of Misoprostol at the community level [81]; construction of mattering waiting room [14], and augmentation of compressive emergency obstetrics care (establishment of mini blood band and expansion of caesarean providing facility) [25, 82]. Despite all these efforts, due to poor utilization of innovative technologies such as the use of uterine balloon tamponade, and simplified dosing of magnesium sulfate [25, 83], postpartum haemorrhage remains unacceptably high in Ethiopia [25]. Overall, the result suggests that there is a huge gap in early detection, response, and treatment of postpartum haemorrhage.

As stated above HDP was also positively related to postpartum maternal death in Ethiopia. The finding was consistent with studies conducted in Ethiopia (Hawwasa, Yirgalem, and Hossana) [84], Nigeria [85], Turkey [86], Ghana [87], and Uganda [88]. The possible justification could be HDP’s strong relation to the delay in seeking care and the delay in initiating anticonvulsant prophylaxis and antihypertensive drugs, which will result in irreversible damage to the brain because of intracranial haemorrhage. This result suggested the need for boosting the health system by providing innovative technologies, which improve proteinuria tests and blood pressure measurements [83]. In combination with efforts to enhance health-seeking behaviour, improving the referral system and availing essential drugs and supplies are required to reduce HDP during the postpartum period [89, 90].

In addition, pregnancy-related infection also has a vital role in maternal death after delivery. This finding was aligned with studies done in 52 countries [91], including Uganda [92], and India [93]. The possible explanations that could contribute to the infection might be related to pre-delivery factors (pre-labour rupture of membranes, prolonged labours, multiple vaginal examinations (more than five), the health status of the women (anaemia, primiparity, and poor nutrition), and clinical procedures during delivery (episiotomy, caesarean section, and other invasive procedures). However, the outcome of the women is determined by the adequacy of vital signs assessment and early administration of antimicrobial therapy. Contrastingly, in the last twenty years, Ethiopia has declined maternal mortality due to infection by practising infection control measures [94]. Generally, the finding indicated that pregnancy-related infection should be the other area of intervention since it is one of the major causes of maternal death in Ethiopia.

Furthermore, Non-obstetrics complication was also positively associated with postpartum maternal death. This finding is in line with studies done in Ethiopia (Jimma) [95] and Nigeria [37, 96]. In Ethiopia, the leading indirect cause of maternal death was severe anaemia unrelated to haemorrhage [25]. The possible explanation for anaemia related death during the postpartum period relates to women’s circulatory decomposition, which is manifested by increased cardiac output and decreased ability to blood loss, which ultimately results in shock and death [97]. On the other hand, the risk factors of anaemia such as low dietary intake of iron, intestinal or blood parasite infection, and chorine illness [98, 99], could be handled during ANC visit by supplementation of iron, folic acid, and deworming.

Failure to recognize the complication of pregnancy is one of the individual factors related to postpartum maternal death. The finding of this study is parallel with studies done in Nigeria [100], Uganda [101], and Somalia [102]. The possible justification might be the fact that poor knowledge of obstetrics complications could make the women less prepared, which may have a negative consequence on accepting appropriate and timely referral to essential obstetric care. Similarly, knowledge of obstetric complications is influenced by educational status, the proximity of health facilities, and previous history of obstetric complications [103, 104]. To address this challenge, Ethiopia introduced a health extension program in 2003, aiming to bring health knowledge and basic care directly to the households at a grassroots level [105]. However, as depicted in the result, further work must be done at the community level.

District-level factors such as scarcity of equipment and supplies and late management after admission were significantly associated with postpartum maternal death. The finding was congruent in case of shortage of equipment and supplies with studies done in Egypt [106], Tanzania [107], and Malawi [108]. This clearly shows that the shortage of blood and blood product is the main challenge in managing obstetrics complications in Ethiopia and other Sub-Saharan African countries [109, 110]. To this effect, the establishment of a mini blood bank at Comprehensive Emergency Obstetric and New-born Care (CEmONC) facilities in Ethiopia was considered as a mitigation plan; despite that, its implementation was challenged by a low blood donation rate, and inadequate testing and quality monitoring capacity [4, 25, 110]. Overall, this result suggests that addressing the gap in availing essential life-saving maternal commodities (medicines, medical devices, and health supplies) should be the target area of intervention to reduce preventable postpartum maternal death.

Finally, late management of women after admission is also one of the district-level factors associated with maternal death after delivery. It is usually framed by a delay in waiting for treatment, which takes more than 30 minutes from the time of admission to assessment and receiving treatment [111]. A similar result was observed in studies done in Ethiopia (Addis Ababa and Tigray) [112, 113], Mozambique [114], Brazil [115], and India [116]. This might be due to the reason for delays such as lack of essential maternal commodities, insufficient training, poor attitudes toward a patient, and poor facility infrastructure related to the operation room and surgical facilities. On the contrary, Ethiopia has designed an alternative strategy to augment the managing capacity of obstetrics emergencies at lower-level health facilities. To this effect, the country has introduced a training program for middle–level health care professionals called the Integrated Emergency Surgical Officers (IESO). Those professionals are trained to handle obstetrics emergencies at lower-level health facilities [117]. In addition, the country has invested resources to upgrade the existing health facilities [118]. However, despite all these efforts, facility-level barriers should still be considered as a milestone to achieve the aspired goal under SDG.

The study has limitations that need to be acknowledged. First, the data used for the analysis was secondary data with single-point time. Thus, only associations were examined, and it was impossible to confirm any causality. Second, all identified, confirmed, and reported maternal death through a weekly reporting system were not reviewed and sent via MDRF to the next level, which might introduce potential bias to the study. Third, nearly all deaths were reported and reviewed from public facilities with limited involvement of private health facilities, and this could affect the representativeness of the study. Fourth, a small number of maternal deaths were captured by the system, which is against national estimates and might compromise the inclusiveness of the study.

## Conclusion

Overall, both individual and facility-level variables were significantly associated with postpartum maternal death among reviewed maternal deaths in Ethiopia. Thus, mothers with previous medical history (history of ANC follow up and party), medical cause (obstetrics haemorrhage, HDP, pregnancy-related infection, and non-obstetrics complication), personal factors (poor knowledge of obstetrics complications), and facility-level barriers (shortage of life-saving maternal commodities and delay in receiving treatment) were at increased risk of maternal death after delivery. The study corroborates that handling individual and district-level factors associated with postpartum maternal death through pragmatic policies and programs is essential to reducing maternal death after delivery. Therefore, emphasis should be given to encouraging the utilization of ANC service with more priority to higher parity women, so that they can get information on obstetric complications, birth preparedness, and complication readiness to prevent delay in seeking care, and improve early diagnosis and treatment of anaemia. Since the two medical causes of death (HDP and obstetrics haemorrhage) were key determinants for postpartum death in Ethiopia, it is better to enhance the application of innovative technologies, which are suitable for resource constraints settings. Accordingly, innovative services such as utilizing uterine balloon tamponade, improving proteinuria testing, and having better blood pressure measurements are recommended. As for the facility level barriers, further improvement in service delivery at health facilities should be the top priority.
